# Barrier Diamond-like Carbon Coatings on Polydimethylsiloxane Substrate

**DOI:** 10.3390/ma15113883

**Published:** 2022-05-29

**Authors:** Witold Kaczorowski, Damian Batory, Witold Szymański, Klaudia Lauk, Jakub Stolarczyk

**Affiliations:** 1 Institute of Material Science and Engineering, Faculty of Mechanical Engineering, Lodz University of Technology, Stefanowskiego 1/15, 90-537 Lodz, Poland; witold.szymanski@p.lodz.pl (W.S.); klaudia.lauk@dokt.p.lodz.pl (K.L.); 212077@edu.p.lodz.pl (J.S.); 2 Department of Vehicles and Fundamentals of Machine Design, Faculty of Mechanical Engineering, Lodz University of Technology, Stefanowskiego 1/15, 90-537 Lodz, Poland; damian.batory@p.lodz.pl

**Keywords:** DLC, surface modification, PDMS, plasma, CVD, Raman spectroscopy

## Abstract

The plasma modification of polydimethylsiloxane (PDMS) substrates is one way to change their surface geometry, which enables the formation of wrinkles. However, these changes are very often accompanied by the process of restoring the hydrophobic properties of the modified material. In this work, the RF PACVD device (radio frequency plasma-assisted chemical vapor deposition) was used, with which the plasma treatment of PDMS substrates was carried out in argon, nitrogen, oxygen, and methane atmospheres at variable negative biases ranging from 100 V to 500 V. The obtained results show the stability of contact angles for deionized water only in the case of surfaces modified by diamond-like carbon (DLC) coatings. The influence of the applied production conditions on the thickness (between 10 and 30 nm) and chemical structure (ID/IG between 0.41 and 0.8) of DLC coatings is discussed. In the case of plasma treatments with other gases introduced into the working chamber, the phenomenon of changing from hydrophilic to hydrophobic properties after the modification processes was observed. The presented results confirm the barrier nature of the DLC coatings produced on the PDMS substrate.

## 1. Introduction

Due to its properties, PDMS is a polymer material used in many applications, ranging from biomedicine to power engineering [1,2,3,4,5]. In addition, the properties of its surface can be changed via plasma modification, including corona plasma, DBD, and RF PACVD [1,4]. Laser treatment and UV radiation are also used for this purpose [1,6,7,8,9]. Obviously, the most promising are those treatments that allow for the uniform modification of the entire surface, among which RF PACVD methods are the most frequently found in the literature [1,4]. Nevertheless, the very application of plasma also influences the aging processes taking place in the outer layer and on the surface itself. In most cases, PDMS plasma treatment involves the problem of a short-term change in the contact angle, sometimes forcing their use as a hydrophilic surface within a few dozen minutes from the end of the modification process. Interestingly, the use of plasma techniques in the modification of polydimethylsiloxane results in the formation of thin, silicon-like coatings (SiOx) on its surface [10,11], which may also play an important role in the aging process [1]. It also turns out that these coatings are an indispensable effect of the use of plasma, and the type of working atmosphere (gases introduced into the processes) is, in most cases, irrelevant [1]. The only exceptions are the processes conducted with use of methane [12,13]. The use of corrugated polymer surfaces with DLC coatings seems to be of particular interest in biological studies on cell proliferation [4,14,15]. The change in contact angle over time, with modified polymeric substrates stored in the air, can be explained in at least three ways. The first of them involves the migration from the inside to the outside of non-cross-linked so-called low molecular weighted (LMW) siloxanes, which slip through the porosities in the aforementioned silicon-like coatings, or through cracks resulting from the mismatch between the mechanical properties of the resulting coatings and the polymer substrate [16,17]. It has even been reported that the occurrence of cracks on the surfaces of SiOx coatings on PDMS may intensify the process of returning to hydrophobic recovery [17]. It is also possible to link the fracture process of silicon-like coatings with the duration of the processes [17]. Generally, it can be summarized that this method of changing the surface contact angle is related to the migration of LMW particles from the center of the material toward the thermally unstable hydrophilic PDMS surface [16,17,18]. An additional, possible effect of the described phenomena is the potential oxidation of the abovementioned molecules in the plasma and their diffusion from the outside into the material [16,18]. The second mechanism is related to the interaction of free radicals and other plasma macromolecules, which may diffuse into the substrate and affect the oxidation processes [16,19]. Interestingly, some free radicals may remain in the polymer network for weeks or months, depending on the processes taking place on the surface [19]. The third mechanism is based on the reorientation of chemical bonds on the surface of polymers [2]. This process is characteristic of larger polymer chains in which the modified part diffuses into the material and the unmodified section diffuses to the surface [16,18]. According to Mortazavi [16], the processes of hydrophobic recovery can be controlled by the parameters of plasma modification, which consist of the gas treatment, modification time, temperature, and humidity of the sample storage environment [16,18,20]. It is also important that it is possible to influence the rate of change of the surface properties of polymers treated with the plasma. Such methods include the processes of cross-linking their surfaces or producing coatings on them [19]. However, as already mentioned, any cracks in the surface of the coatings formed on PDMS represent a potential site for LOW migration, leading to hydrophobic surface recovery [1]. Another way to protect the surface of polymers is to create a DLC coating, which, apart from stabilizing the contact angle, can also improve a number of other surface properties, such as tribological or biological [21,22,23]. It is worth emphasizing that this type of coating is known as an excellent barrier against the transfer of metal ions in biomedical applications [24,25]. DLC coatings are simply a mixture of sp^3^, sp^2^ hybridized carbon atoms, and hydrogen. Their properties, which can be varied in a very wide range, depend on the presented composition and chemical structure. Of course, in the case of carbon coatings produced on polymer substrates, the range of their properties is limited by the thermal resistance of the substrates themselves. Nevertheless, works on the use of carbon coatings are carried out both in the case of hard, wear-resistant polymers, such as PEEK [22], and those that are “soft”, such as PDMS [26]. The use of DLC coatings on substrates such as PDMS is inevitably combined with the appearance of wrinkles on the surface of polymers. It is an issue that has been widely described in the literature [10,27,28,29,30,31,32]. Most often, wrinkles on modified PDMS surfaces are described as the result of mismatching the elasticity modulus of the coating and the polymer substrate [26,27,31]. However, when analyzing this issue in more detail, it is worth noting that the formation of wrinkles is the effect of mismatching the deformation of the formed coating when the compressive stress exceeds the threshold value [29,30,32]. There are a number of mathematical models describing the wavelength and amplitude of the wrinkles, depending on the material properties of the substrate, the coating, and even the intermediate layer that may be formed during the modification processes [27]. Changes in the mentioned parameters, which, in fact, perfectly describe the surface geometry, are often reflected in changes in the value of surface free energy [27]. Wrinkles on the surface of DLC coatings produced on the PDMS substrate may also be the result of the applied additional tensile stresses [10,33]; however, in this case, cracks in the coating very often occur. On the other hand, the application of tests with a variable tensile force of DLC coatings on PDMS substrates allows us to know other important properties of such a composite, e.g., coating adhesion to the substrate or the presence of a cross-linked substrate interlayer [34]. Similar relationships also apply to other coatings on PDMS substrates, such as SiOx, gold [27,33], silver, molybdenum [29], or polymer coatings [28]. As already described in our previous work regarding DLC coatings on PDMS, substrates deposited by the RF PACVD method are uniform and crack-free. They increase the stiffness modulus of elasticity and improve the tribological properties (reduce the friction coefficient and wear rate) of the PDMS surface. The adhesion of DLC coatings to PDMS substrates increases with the increasing negative autopolarization potential [34]. In this paper, the authors focused on the analysis of the influence of various atmospheres and energy parameters of radio frequency plasma on changes in the contact angle for deionized water and parameters describing the modified surface. Additionally, in the case of PDMS substrates modified in methane plasma, the analysis of the chemical structure of the coatings was carried out using Raman spectroscopy. The presented studies are of particular importance for further research on the development of the optimal surfaces for cell cultures for biomedicine.

## 2. Materials and Methods

### 2.1. Manufacturing of PDMS Substrates

Sylgard 184 polydimethylsiloxane (PDMS) samples from Dow Corning company (Midland, MI, USA) were prepared by hand mixing the polymer base and hardener in a mass ratio of 10: 1, adequate for the presented materials. After degassing in a vacuum chamber, the liquid polymer was poured into Petri dishes in such an amount that the thickness of the obtained samples was 2 mm. Curing of PDMS was carried out at 80 °C for 12 h. From the material prepared in this way, samples with a size of 20 × 20 mm were cut for further research.

### 2.2. Apparatus and Modification Parameters

Modifications of PDMS substrates were carried out in the RF PACVD system described in our previous publications. All processes were carried out with variable negative autopolarization potential (Vb) from 100 V to 500 V with the use of gases such as oxygen, argon, nitrogen, and methane. The duration of the processes was identical for each modification and amounted to 60 s. The most important parameters of the processes are presented in Table 1.

### 2.3. Contact Angle Measurement

The contact angle measurements were carried out with use of Kruss Easy Drop DSA15B device (KRÜSS GmbH, Hamburg, Germany) using deionized water at 25 °C. Each drop placed on the modified surface had a volume of 0.8 µL. The presented test results are the average of 3 measurements of both the left and right contact angle of the deionized water drop.

### 2.4. Atomic Force Microscopy AFM

The surface geometrical structure was measured using Bruker MultiMode V atomic force microscope (Bruker Corporation, Billerica, MA, USA), operating in the tapping mode and equipped with OTESPA scanning probe with a nominal resonance frequency of 300 kHz and an elasticity constant of 26 N/m. Scans of 10 × 10 µm (512 lines) were performed at two randomly selected locations for each of the samples. The obtained scans of the surface were pre-processed in the NanoScope Analysis software, whereas further processing and analysis were carried out with the use of the MountainsMap Premium 5.0 (Version 5, Digital Surf, Besancon, France). For each surface scan, 512 surface profiles were distinguished, which were used to determine the selected roughness parameters Ra and Rz. The amplitude (A) and wavelength (λ) of the ripples were measured manually. For each modification and scan, four profiles were distinguished, and three measurements of the measured value were made. As the extracted wrinkle wave profiles do not reflect the ideal sin (x) function, the amplitude was calculated from three measurements above and three below the mean line. In summation, the mean amplitude was calculated on the basis of 48 measurements, and the average wavelength was calculated on the basis of 24 measurements for each modification.

### 2.5. Raman Spectroscopy

Raman spectroscopy studies were performed on a Renishaw inVia instrument (Gloucestershire, UK). A laser with a wavelength of 532 nm and a 50× lens was used for the tests. In order to analyze changes in the chemical structure of modified or not modified PDMS substrates, spectral ranges from 100 to 3200 cm^−1^ were used, while the analysis of the chemical structure of DLC coatings was carried out based on the range from 900 to 2000 cm^−1^. The obtained Raman spectra for DLC coatings were deconvolved into 4 characteristic peaks in accordance with the literature data [35,36], using the PEAKFIT 4.12 software (Version 4.12, Seasolve, San Jose, CA, USA).

### 2.6. Thickness Analysis

The thicknesses of the DLC coatings were determined only on the reference substrates, which were 513 µm thick <100> silicon wafers. Before the modification processes, each time, in addition to the PDMS substrate, a fragment of a silicon wafer with one half covered was inserted into the reactor chamber. In this way, after the DLC coatings production processes, it was possible to determine the thickness of the coatings using a profilometer and XRR measurements. In the case of testing the thickness profiles, the Hommel Tester T-1000 profilometer (JENOPTIK Industrial metrology, Villingen-Schwenningen, Germany) was used. Additionally, the coatings obtained on the silicon samples were tested by the X-ray reflectivity method, in which the thickness of the coatings was determined based on periodicity of the registered XRR fringes. The Empyrean diffractometer (Malvern Panalytical, Malvern, Worcestershire, UK) working with Cu Kα radiation (λ = 0.15418 nm) was used to obtain the reflectometric curves. The obtained data were processed using X’Pert Reflectivity software (version 4.7, Malvern Panalytical, Malvern, UK).

## 3. Results and Discussion

### 3.1. Changes in Contact Angles over Time of the Modified PDMS Substrates

**Figure 1:**
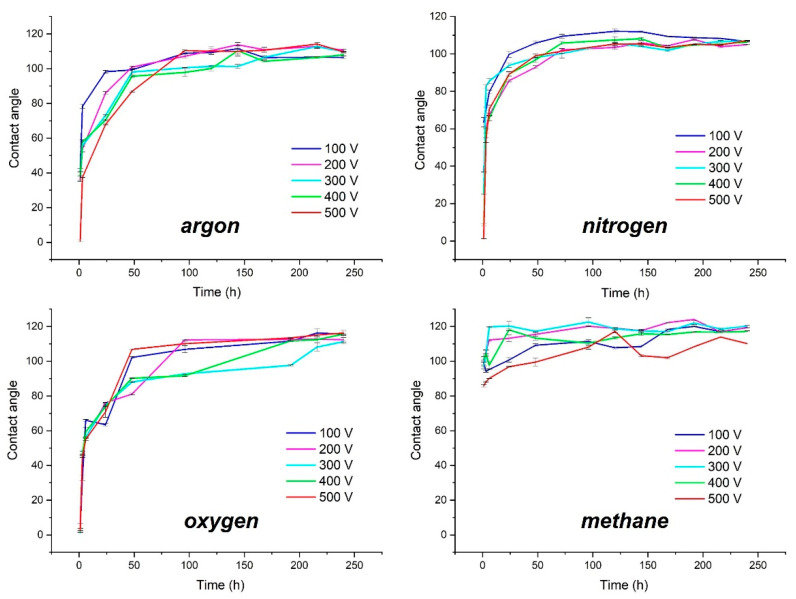
Changes in contact angles over time of the modified PDMS substrates.

The study of changes in the contact angles of the radio frequency plasma modified PDMS surfaces was carried out with the use of deionized water. The analysis covered the influence of the type of plasma working atmosphere (argon, nitrogen, oxygen, and methane) and the values of the negative autopolarization potential, which varied from 100 V to 500 V (Figure 1).

As shown in Figure 1, most of the gases used for the modification processes have a similar effect on the wettability of the modified surface. The exception is methane, for which the contact angle values are almost constant over time. This is related to the production of carbon coatings on the PDMS substrate during plasma processes involving CH_4_, which will be proved in the next subsection. The obtained results confirm that, in this case, we are dealing with the production of barrier coatings that do not allow the LMW to migrate from the inside of the material to the surface, which allows us to observe stable values of contact angles during the entire test cycle. Such properties of DLC coatings are also described in the literature in the case of metal substrates [24,25].

Comparing the course of changes in the contact angles depending on the applied negative autopolarization potential, it can be seen that for argon plasma, the higher the Vb value, the slower the rate of changes on the surface causing the return to hydrophobic properties (especially up to the first 75 h of tests). In the case of nitrogen plasma, these trends are similar, but the scatter of results between modifications at different negative potentials is much smaller than in the case of argon plasma. However, for the oxygen plasma, the relations presented above are not visible. In that case, we observe a different growth rate of the contact angles, depending on the applied Vb. The lowest of them is characteristic of negative potential equal to 300 V, which may prove that, in the case of this sample, under the applied plasma treatment conditions, a thin oxide layer was probably formed on the surface. Our previous studies involving the use of XPS have already shown that there is such a possibility [13]. In the case of using methane plasma for the modification of PDMS substrates, the obtained values of the contact angles are related to the applied negative autopolarization potential. The lowest values were obtained for the substrates modified at Vb = 500 V and then for Vb = 100 V, 400 V, 200 V, and 300 V. This order is not accidental, and, as further tests will show, it is related to the thickness of the DLC coatings produced on the PDMS surface. The thinner the coating, the lower the resulting contact angles are. However, it is worth emphasizing that even in the case of the thinnest coatings, the registered contact angles oscillate around one similar value during the entire measurement. The effect of changes in the contact angle under the influence of thickness may be related to the phenomena occurring during the modification process of PDMS substrates. The literature’s data indicate that in the case of this treatment, the indispensable effect is the incorporation of silicon into DLC coatings [13,37]. Therefore, there is a high probability that the amount of embedded silicon in the DLC structure also depends on the duration of the DLC coatings’ manufacturing process, which decreases with an increasing coating thickness. Additionally, the literature reports that higher silicon content in DLC coatings (usually above a dozen or so percent) results in a contact angle reduction that is even below the value for unmodified DLC coatings [38,39,40]. Obviously, this is one of the possible hypotheses that may be proven in future research. According to the literature, the value of the contact angle of DLC coatings is influenced not only by their chemical structure (C=C sp^2^/C-C sp^3^ ratio) [41] but also by their surface termination (whether they are hydrogen or oxygen terminated) [42,43]. For example, hydrophobic properties can be achieved via hydrogen termination due to the formation of hydrophobic C-H bonds (or, assuming silicon-incorporated DLC, also Si-H bonds) [44]. Thus, the surface termination during or after the deposition process plays an important role in controlling the wettability of the manufactured coatings.

### 3.2. Geometrical Structure Analysis of the Modified PDMS Surfaces

**Figure 2:**
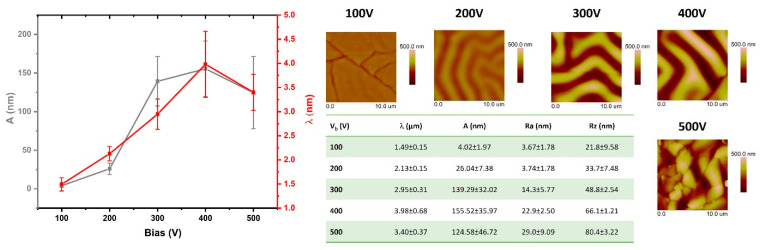
The analysis of changes of surface geometrical structure parameters of PDMS substrates modified in argon plasma.

**Figure 3:**
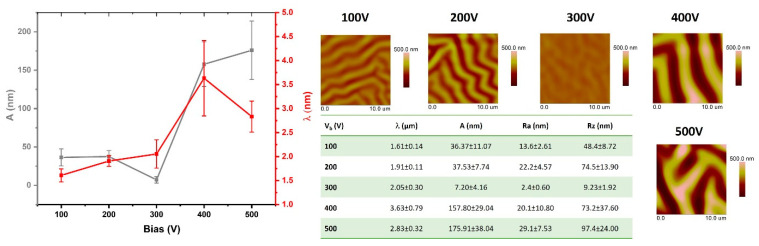
The analysis of chcanges of surface geometrical structure parameters of PDMS substrates modified in nitrogen plasma.

**Figure 4:**
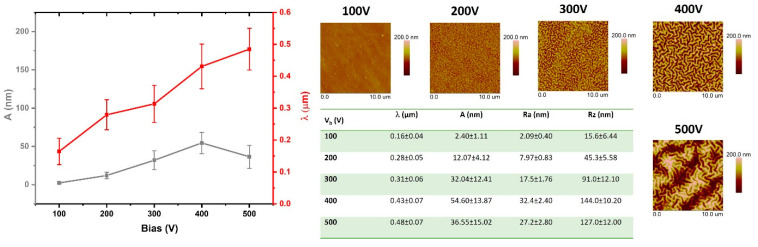
The analysis of changes of surface geometrical structure parameters of PDMS substrates modified in oxygen plasma.

**Figure 5:**
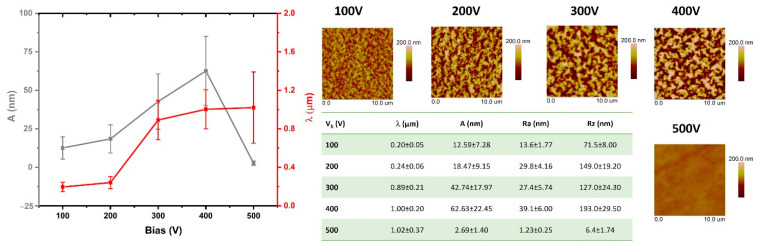
The analysis of changes of surface geometrical structure parameters of PDMS substrates modified in methane plasma.

The test results presented in this section are based on the analysis of AFM images obtained from the modified surfaces. The comparisons include both the geometric parameters of the wrinkles formed on the PDMS surfaces and the surface roughness parameters. Figure 2, Figure 3, Figure 4 and Figure 5 show the comparisons of the working atmosphere and the parameters Vb. The presented results prove that the change in the negative self-bias potential influences changes in the surface geometry of the modified PDMS substrates. This effect is also known from the literature [11,45,46]. As other papers indicate [14,46,47], wrinkles formed on polymers may have a hierarchical character (i.e., two separate profile runs related to the modification in the micro and nanoscale can be distinguished). In our case, such an effect is observed only for the modification of the oxygen plasma using Vb = 500 V. For this modification, the AFM image (Figure 4) shows an additional effect of the changes in the observed wrinkles on a micro scale, and it is possible to determine the geometrical structure parameters for this wave. The value of λ is about 4 µm, and A is about 230 nm. Among all modifications, the lowest values of the obtained wavelength were observed for processes conducted with the use of oxygen plasma. Interestingly, similar results were reported by Gu et al. [45], although they conducted the modifications at low power ranges of up to 60 W. The highest wavelength values were characteristic of the argon atmosphere, which, as indicated in the literature [45], is related to the greater penetration depth of argon molecules due to their smaller size and greater weight than molecules formed, e.g., in nitrogen or oxygen plasma, as well as positive charge (opposite to the negative charge of oxygen or nitrogen ions). By analyzing the obtained values of parameters, namely λ, A, Ra, and Rz, it can be seen that the latter are closely related to the obtained amplitude values. If the value of A increases, the roughness parameters also increase, which is confirmed by the tabular data in Figure 2, Figure 3, Figure 4 and Figure 5.

**Figure 6:**
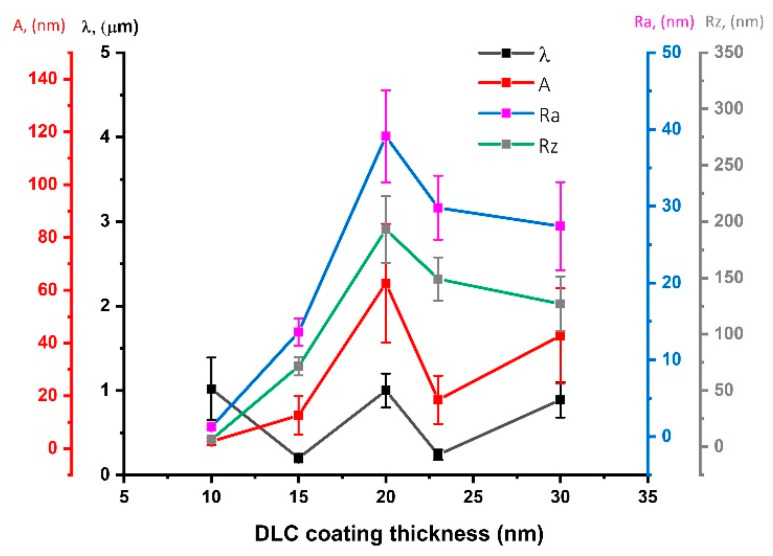
The analysis of changes of surface geometrical structure parameters vs. thickness of the deposited DLC coatings.

When analyzing changes in the geometric structure of PDMS substrates modified in methane plasma, it can be observed that with the increase of the negative self-bias potential up to 400 V, all parameters (λ, A, Ra, Rz) increase. On the other hand, for the sample modified at Vb = 500 V, a drastic decrease in the amplitude value and related roughness parameters can be observed. This effect is closely related to the thickness of the DLC coating deposited on the surface with these parameters, which can be clearly seen in Figure 6. In the range from 0 to 14 nm, with the increasing thickness of the coatings, the value of the obtained amplitude of wrinkles, as well as the surface roughness parameters, increase. For the thickness of 14 nm, we observe the maximum values for all parameters describing the surface geometry of the modified samples. Looking at the dispersion of the standard deviation of the measurements, it can be assumed that, from that moment, some stabilization of surface geometry changes occurs, which seems to be consistent with the results presented in the literature [48]. Generally, the presented data indicate a lack of correlation between the results of the surface roughness and contact angle.

### 3.3. Chemical Structure Analysis of the Modified PDMS Substrates

The chemical structure of the modified PDMS substrates was analyzed based on the results of Raman spectroscopy. Figure S1 shows the Raman spectrum for the unmodified PDMS substrate. According to the literature [49,50], we can distinguish the following characteristic peaks: Si-O-Si symmetric stretching (488 cm^−1^), C-Si stretching vibrations (617 cm^−1^) Si-CH_3_ symmetric rocking (687 cm^−1^), Si-C symmetric stretching (708 cm^−1^), CH_3_ asymmetric rocking + Si-C asymmetric stretching (787 cm^−1^), CH_3_ symmetric rocking (862 cm^−1^), CH_3_ symmetric bending (1262 cm^−1^), CH_3_ asymmetric bending (1412 cm^−1^), CH_3_ symmetric stretching (2907 cm^−1^), and CH_3_ asymmetric stretching (2965 cm^−1^). All PDMS plasma-modified substrates were also subjected to Raman spectroscopy. On the basis of the obtained results, it can be concluded that after the application of the plasma treatment, in most cases (excluding the methane atmosphere), no significant changes in the location and intensity of the characteristic peaks were found. In the additional materials are presented the spectra ranging from 400 to 1500 cm^−1^ for PDMS modification in oxygen, argon, and nitrogen plasma using the negative autopolarization potential of 300 V, i.e., for the potential at which the obtained contact angles in most cases had the lowest values (Figures S2–S4).

**Figure 7:**
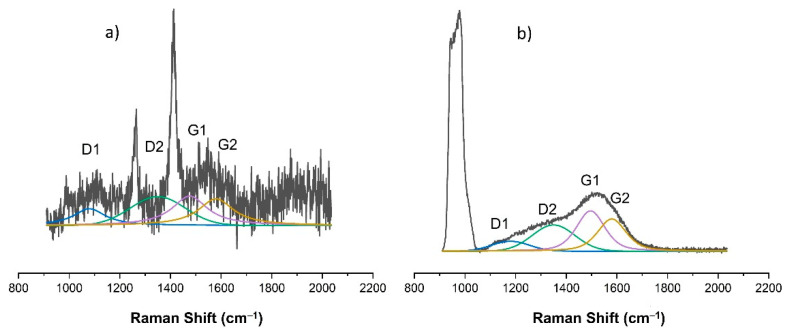
Raman spectra of DLC coating deposited using methane plasma under the negative self-bias Vb = 100 V on: (**a**) PDMS substrate; (**b**) silicon wafer.

**Figure 8:**
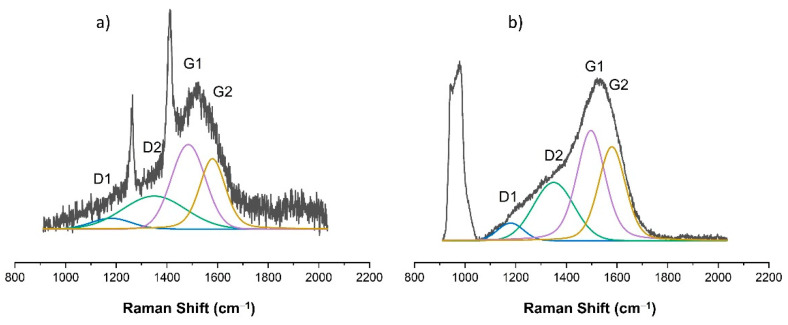
Raman spectra of DLC coating deposited using methane plasma under the negative self-bias Vb = 200 V on: (**a**) PDMS substrate; (**b**) silicon wafer.

**Figure 9:**
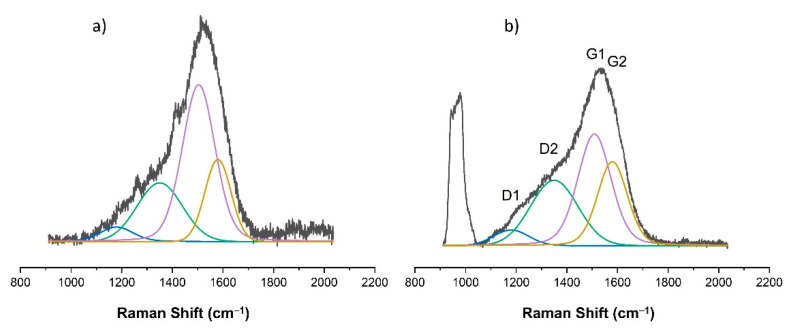
Raman spectra of DLC coating deposited using methane plasma under the negative self-bias Vb = 300 V on: (**a**) PDMS substrate; (**b**) silicon wafer.

**Figure 10:**
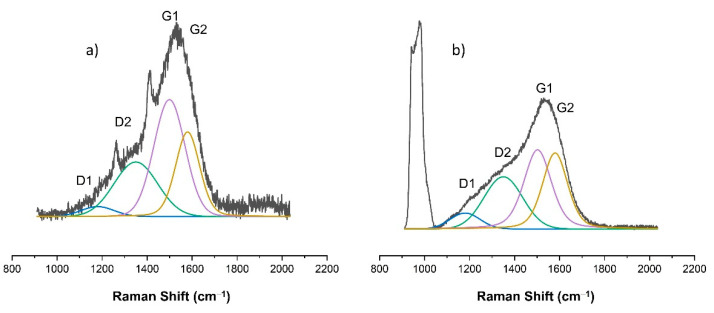
Raman spectra of DLC coating deposited using methane plasma under the negative self-bias Vb = 400 V on: (**a**) PDMS substrate; (**b**) silicon wafer.

**Figure 11:**
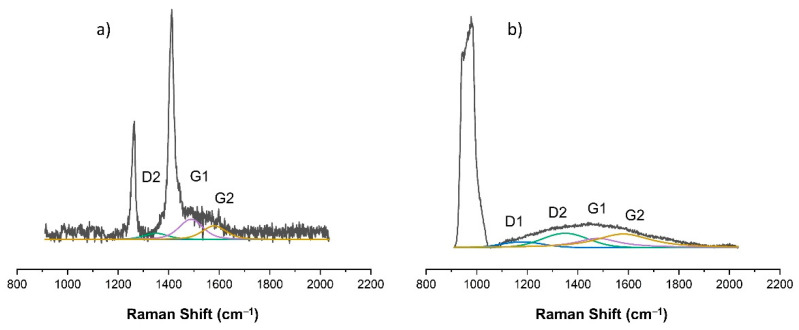
Raman spectra of DLC coating deposited using methane plasma under the negative self-bias Vb = 500 V on: (**a**) PDMS substrate; (**b**) silicon wafer.

As already indicated, changes in the chemical structure of the modified PDMS substrates were observed for modifications made with the use of methane plasma. Figure 7, Figure 8, Figure 9, Figure 10 and Figure 11 show the Raman spectra ranging from 900 cm^−1^ to 2000 cm^−1^ of both the PDMS modified in methane plasma with different negative autopolarization potentials and the reference material, silicon. These results are evidence of the formation of carbon coatings on PDMS substrates; therefore, further analysis was undertaken based on a model assuming curve fitting with four characteristic peaks: D1, D2, G1, and G2, in accordance with the literature [35,36]. Based on the analysis of the areas of the determined characteristic peaks, the ID/IG ratio for the obtained spectra was determined, in our case, to be equal to the expression (ID1 + ID2)/(IG1 + IG2). The results are included in Table 2. Both the obtained Raman spectra trend and the ID/IG ratios are consistent with the literature data regarding the production of DLC coatings on polymer substrates [51,52]. On the basis of the presented results and the literature review [53,54,55], it can be stated that when using Vb equal to 300 V, DLC coatings with the highest content of C-C sp^3^ hybridized bonds were obtained, since the lowest values of ID/IG ratio were obtained for these coatings. On the other hand, the use of a negative polarization potential at the level of 100 V resulted in the formation of a DLC coating with the highest content of C = C sp^2^ hybridized bonds. Comparing the data for PDMS and silicon substrates included in Table 2 similar trends regarding the changes in the chemical structure can be observed. However, the data cannot be compared with each other because the chemical structure of DLC coatings and their other properties are determined by the substrate material [56,57]. Other researchers obtained similar results regarding silicon and PDMS substrates [36,51]. Interestingly, the obtained Raman spectra also provide information on the thickness of the produced DLC coatings. The smaller the thickness of the deposited coating, the greater the intensity of the peaks originating from the substrate. Therefore, for PDMS substrates, it is possible to correlate the Raman spectra for DLC with the intensity of CH_3_ bonds at 1262 cm^−1^ and 1412 cm^−1^, while silicon substrates with bands originating from bonds at approx. 935–990 cm^−1^ can be derived from multi-phonon scattering from the silicon substrates [58]. On this basis, it can be concluded that the thickest coatings were produced at Vb equal to 300 V, and the thinnest ones at Vb equal to 500 V.

### 3.4. Determination of Thickness of DLC Coatings in the PDMS Substrates

The thicknesses of the produced carbon coatings on the PDMS substrates were determined indirectly using silicon substrates placed in the reactor chamber together with the polymer material. Based on the profilometer and XRR measurements, it was determined that the thickness of synthesized DLC coatings ranged from 10 to about 30 nm (Table 3), with the thickest coating produced during a 1 min process at Vb = 300 V. The obtained results are consistent with the observations described in the paragraph related to the Raman spectroscopy results. Certain relationships can be found between the thickness of the coatings and the obtained contact angles. The lowest values of the contact angles were observed for the thinnest coatings. On the other hand, the highest values of the contact angles were characteristic of the thickest DLC coatings. As described in Section 3.1, this may be related to the amount of silicon built into the structures of the carbon coatings during the deposition processes.

## 4. Conclusions

The presented paper shows the properties of PDMS surfaces modified with the use of four different gases. As shown by the results of the changes in the contact angles for deionized water over time, it is only possible for the surfaces to be stabilized via methane plasma. This type of plasma determines the formation of a DLC coating on the surface of the modified polymer, which is an excellent barrier that prevents the migration of LWMs (low molecular weighted (LMW) siloxanes) from inside the modified PDMS substrates. Interestingly, the obtained values of the contact angles for the PDMS surfaces after modification with CH_4_ seem to correlate with the thickness and chemical structure of the produced DLC coatings. The lower the thickness of the coating produced on the PDMS surfaces, the lower the values of the contact angles registered for deionized water in the wettability tests. These results could be related to the chemical composition of the DLC coatings on PDMS substrates, especially in regard to the level of Si coming from the modified polymer substrate and being incorporated into the amorphous carbon structure. Coatings of the lowest thickness may contain the highest content of silicon, which determines the presence of a greater number of Si-Ox bonds on the surface and explains their higher hydrophilicity. The higher the content of C-C sp^3^ hybridized bonds in the coatings (low ID/IG intensity ratio determined from the Raman spectra), the higher the contact angle values registered for the DLC coatings. Both the thickness and the chemical structure of the produced carbon coatings can be controlled by the negative autopolarization potential. All the performed plasma modifications changed the geometrical structures of the PDMS surfaces. The processes of wrinkle formation on the PDMS surfaces can be controlled by selecting the plasma’s working atmosphere and its energy parameters.

## Figures and Tables

**Table 1:** Parameters of modification processes.

Gas	Gas Flow (sccm)	Pressure (Pa)	Bias Vb (V)	Power P (W)	Time (s)
**Ar**	60	29	100	92	60
			200	140	
			300	280	
			400	460	
			500	690	
**N_2_**		27	100	84	
			200	136	
			300	250	
			400	420	
			500	570	
**O_2_**		30	100	82	
			200	125	
			300	220	
			400	350	
			500	550	
**CH_4_**		33	100	80	
			200	120	
			300	210	
			400	340	
			500	540	

**Table 2:** ID/IG intensity ratio of DLC coatings synthesized on silicon and PDMS substrates.

Bias (V)	ID/IG	
	Silicon	PDMS
100	0.59	0.8
200	0.54	0.5
300	0.53	0.41
400	0.52	0.45
500	0.56	0.50

**Table 3:** The average thickness of DLC coatings deposited on silicon substrates, determined via profilometry and XRR methods.

	Negative Self-Bias [V]				
	100	200	300	400	500
Average thickness of DLC coating on silicon substrates [nm]	15 ± 5	23 ± 4	30 ± 6	20 ± 3	10 ± 3

## Data Availability

The data used to support the findings of this study are available from the corresponding author upon request.

## Supplementary Materials

The following supporting information can be downloaded at: https://www.mdpi.com/article/10.3390/ma15113883/s1, Figure S1: Raman spectra of PDMS substrates modified in argon plasma under the negative self-bias Vb = 300 V. Figure S2: Raman spectra of PDMS substrates modified in nitrogen plasma under the negative self-bias Vb = 300 V. Figure S3: Raman spectra of PDMS substrates modified in oxygen plasma under the negative self-bias Vb = 300 V. Figure S4: Raman spectra of PDMS substrates modified in oxygen plasma under the negative self-bias Vb = 300 V.

## References

[1] Wang Z., Lou Y., Zheng F., Zhang N., Yin C., Li J., He C., Peng X., Huang Z., Fang P. (2018). Study on surface structure of plasma-treated polydimethylsiloxane (PDMS) elastomer by slow positron beam. Surf. Interface Anal..

[2] Wolf M.P., Salieb-Beugelaar G.B., Hunziker P. (2018). PDMS with designer functionalities-Properties, modifications strategies, and applications. Prog. Polym. Sci..

[3] Zahid A., Dai B., Hong R., Zhang D. (2017). Optical properties study of silicone polymer PDMS substrate surfaces modified by plasma treatment. Mater. Res. Express.

[4] Ban M., Tobe S., Takeuchi L. (2018). Effects of diamond-like carbon thin film and wrinkle microstructure on cell proliferation. Diam. Relat. Mater..

[5] Cheng X., Meng B., Chen X., Han M., Chen H., Su Z., Shi M., Zhang H. (2016). Single-step fluorocarbon plasma treatment-induced wrinkle structure for high-performance triboelectric nanogenerator. Small.

[6] Gokaltun A., Yarmush M.L., Asatekin A., Usta O.B. (2017). Recent advances in nonbiofouling PDMS surface modification strategies applicable to microfluidic technology. Technology (Singap. World Sci.).

[7] Qi L., Ruck C., Spychalski G., King B., Wu B., Zhao Y. (2018). Writing wrinkles on poly(dimethylsiloxane) (PDMS) by surface oxidation with a CO2 laser engraver. ACS Appl. Mater. Interfaces.

[8] Leigh B.L., Cheng E., Xu L., Derk A., Hansen M.R., Guymon C.A. (2019). Antifouling photograftable zwitterionic coatings on PDMS substrates. Langmuir.

[9] Wang Q., Sun G., Tong Q., Yang W., Hao W. (2021). Fluorine-free superhydrophobic coatings from polydimethylsiloxane for sustainable chemical engineering: Preparation methods and applications. Chem. Eng. J..

[10] Ban M., Hagiwara T., Masumoto Y. (2017). Partial formation of linear concavo-convex microstructure onto microwells by diamond-like carbon thin film deposition. Diam. Relat. Mater..

[11] Glatz B.A., Fery A. (2019). The influence of plasma treatment on the elasticity of the in situ oxidized gradient layer in PDMS: Towards crack-free wrinkling. Soft Matter.

[12] Lee D., Yang S. (2012). Surface modification of PDMS by atmospheric-pressure plasma-enhanced chemical vapor deposition and analysis of long-lasting surface hydrophilicity. Sens. Actuat. B Chem..

[13] Kaczorowski W., Szymański W., Batory D., Niedzielski P. (2015). Effect of plasma treatment on the surface properties of polydimethylsiloxane. J. Appl. Polym. Sci..

[14] Ma L., He L., Ni Y. (2020). Tunable hierarchical wrinkling: From models to applications. J. Appl. Phys..

[15] Zhou Q., Kuhn P.T., Huisman T., Nieboer E., van Zwol C., van Kooten T., van Rijn P. (2015). Directional nanotopographic gradients: A high-throughput screening platform for cell contact guidance. Sci. Rep..

[16] Mortazavi M., Nosonovsky M. (2012). A model for diffusion-driven hydrophobic recovery in plasma treated polymers. Appl. Surf. Sci..

[17] Kim J., Chaudhury M.K., Owen M.J., Orbeck T. (2001). The mechanisms of hydrophobic recovery of polydimethylsiloxane elastomers exposed to partial electrical discharges. J. Colloid Interface Sci..

[18] Bacharouche J., Haidara H., Kunemann P., Vallat M.F., Roucoules V. (2013). Singularities in hydrophobic recovery of plasma treated polydimethylsiloxane surfaces under non-contaminant atmosphere. Sens. Actuator A Phys..

[19] Vandenbossche M., Hegemann D. (2018). Recent approaches to reduce aging phenomena in oxygen- and nitrogen-containing plasma polymer films: An overview. Curr. Opin. Solid State Mater. Sci..

[20] Pascual M., Kerdraon M., Rezard Q., Jullien M.C., Champougny L. (2019). Wettability patterning in microfluidic devices using thermally-enhanced hydrophobic recovery of PDMS. Soft Matter.

[21] Kaczorowski W., Batory B., Szymański W., Kaźmierczak T., Kotela I., Niedzielski P. (2016). Frictional behavior of polyurethane modified by carbon coatings synthesized in dual-frequency plasma. Tribol. T..

[22] Kaczorowski W., Batory B., Szymański W., Niedzielski P. (2015). Evaluation of the surface properties of PEEK substrate after two-step plasma modification: Etching and deposition of DLC coatings. Surf. Coat. Technol..

[23] Kaczorowski W., Batory D., Jakubowski W., Szymański W., Komorowski P., Walkowiak B., Sanak M., Niedzielski P. (2016). Physicochemical and Biological Investigation of Different Structures of Carbon Coatings Deposited onto Polyurethane. Braz. Arch. Biol. Technol..

[24] Grill A. (2003). Diamond-like carbon coatings as biocompatible materials—An overview. Diam. Relat. Mater..

[25] Sui J.H., Cai W. (2006). Effect of diamond-like carbon (DLC) on the properties of the NiTi alloys. Diam. Relat. Mater..

[26] Alam M.S., Mukherjee N., Ahmed S.F. (2018). Electron field emission property of nanostructure wrinkle thin film induced by amorphous diamond like carbon. Mater. Today Proc..

[27] Teixeira F.S., Araujo W.W.R., Gushiken N.K., Cattani M., Salvadori M.C. (2017). On the influence of PDMS (polydimethylsiloxane) substrate surface energy in wrinkling of DLC (diamond-like carbon) thin films. J. Appl. Phys..

[28] Ferreira S., Piedade A.P. (2020). Influence of extracellular mimicked hierarchical nano-micro-topography on the bacteria/abiotic interface. Polymers.

[29] Li S.J., Wu K., Yuan H.Z., Zhang J.Y., Liu G., Sun J. (2019). Formation of wrinkled patterns in metal films deposited on elastic substrates: Tunability and wettability. Surf. Coat. Technol..

[30] Wang Y., Xiao J. (2017). Programmable, reversible and repeatable wrinkling of shape memory polymer thin films on elastomeric substrates for smart adhesion. Soft Matter.

[31] Lee W.K., Engel C.J., Huntington M.D., Hu J., Odom T.W. (2015). Controlled three-dimensional hierarchical structuring by memory-based, sequential wrinkling. Nano Lett..

[32] Vandeparre H., Leodpoldes J., Poulard C., Desprez S., Derue G., Gay C., Damman P. (2007). Slippery or sticky boundary conditions: Control of wrinkling in metal-capped thin polymer films by selective adhesion to substrates. Phys. Rev. Lett..

[33] Lee J.S., Hong H., Park S.J., Lee S.J., Kim D.S. (2017). A simple fabrication process for stepwise gradient wrinkle pattern with spatially-controlled wavelength based on sequential oxygen plasma treatment. Microelectron. Eng..

[34] Kaczorowski W., Gajewski K., Szymanski W., Batory D., Wojciechowska A., Swiatek L., Gotszalk T., Niedzielski P. (2018). Evaluation of mechanical properties of carbon coatings synthesised in radio frequency plasma on PDMS. Surf. Coat. Technol..

[35] Tai F.C., Lee S.C., Chen J., Wei C., Chang S.H. (2009). multipeak fitting analysis of raman spectra on DLCH film. J. Raman Spectrosc..

[36] Kaczorowski W., Świątek H., Łuczak K., Głuszek M., Cłapa M. (2021). Impact of plasma pre-treatment on the tribological properties of DLC coatings on PDMS substrates. Materials.

[37] Nagashima S., Hasebe T., Tsuya D., Horikosh T., Ochiai M., Tanigawa S., Koide Y., Hotta A., Suzuki T. (2012). Controlled formation of wrinkled diamond-like carbon (DLC) film on grooved poly(dimethylsiloxane) substrate. Diam. Relat. Mater..

[38] Bociaga D., Kaminska M., Sobczyk-Guzenda A., Jastrzebski K., Swiatek L., Olejnik A. (2016). Surface properties and biological behaviour of Si-DLC coatings fabricated by a multi-target DC–RF magnetron sputtering method for medical applications. Diam. Relat. Mater..

[39] Ogwu A.A., Okpalugo T.I.T., Ali N., Maguire P.D., McLaughlin J.A.D. (2008). Endothelial cell growth on silicon modified hydrogenated amorphous carbon thin films. J. Biomed. Mater. Res. B.

[40] Okpalugo T.I.T., Ogwu A.A., Maguire P.D., McLaughlin J.A.D. (2004). Platelet adhesion on silicon modified hydrogenated amorphous carbon films. Biomaterials.

[41] Paul R., Das S.N., Dalui S., Gayen R.N., Roy R.K., Bhar R., Pal A.K. (2008). Synthesis of DLC films with different sp2/sp3 ratios and their hydrophobic behaviour. J. Phys. D Appl. Phys..

[42] Salvadori M.C., Araújo W.W.R., Teixeira F.S., Cattani M., Pasquarelli A., Oks E.M., Brown I.G. (2010). Termination of diamond surfaces with hydrogen, oxygen and fluorine using a small, simple plasma gun. Diam. Relat. Mater..

[43] Artemenko A., Ižák T., Marton M., Ukraintsev E., Stuchlík J., Hruška K., Vojs M., Kromka A. (2019). Stability of the surface termination of nanocrystalline diamond and diamond-like carbon films exposed to open air conditions. Diam. Relat. Mater..

[44] Sasaki Y., Osanai H., Ohtani Y., Murono Y., Sato M., Kobayashi Y., Enta Y., Suzuki Y., Nakazawa H. (2022). Influence of hydrogen gas flow ratio on the properties of silicon- and nitrogen-doped diamond-like carbon films by plasma-enhanced chemical vapor deposition. Diam. Relat. Mater..

[45] Gu B., Ko D., Jo S., Hyun D.C., Oh H.J., Kim J. (2020). Effect of low-pressure plasma treatment parameters on wrinkle features. Materials.

[46] Miao L., Cheng X., Chen H., Song Y., Guo H., Zhang J., Chen X., Zhang H. (2018). Fabrication of controlled hierarchical wrinkle structures on polydimethylsiloxane via one-step C4F8 plasma treatment. J. Micromech. Microeng..

[47] Xu H., Shi T., Liao G., Xia Q. (2019). Controlling nested wrinkle morphology through the boundary effect on narrow-band thin films. Front. Mech. Eng..

[48] Rahmawan Y., Moon M.W., Kim K.S., Lee K.R., Suh K.Y. (2010). Wrinkled, dual-scale structures of diamond-like carbon (DLC) for superhydrophobicity. Langmuir.

[49] Bae S.C., Lee H., Lin Z., Granick S. (2005). Chemical imaging in a surface forces apparatus: Confocal raman spectroscopy of confined poly(dimethylsiloxane). Langmuir.

[50] Tomar B.S., Shahin A., Tirumkudulu M.S. (2020). Cracking in drying films of polymer solutions. Soft Matter.

[51] Ahmed S.F., Nagashima S., Lee J.Y., Lee K.R., Kim K.S., Moon M.W. (2014). Self-assembled folding of a biaxially compressed film on a compliant substrate. Carbon.

[52] Ahmed S.F., Nho G.H., Lee K.R., Vaziri A., Moon M.W. (2010). High aspect ratio wrinkles on a soft polymer. Soft Matter.

[53] Foong Y.M., Hsieh J., Li X., Chua D.H.C. (2009). The study on the effect of erbium on diamond-like carbon deposited by pulsed laser deposition technique. J. Appl. Phys..

[54] Batory D., Jedrzejczak A., Kaczorowski W., Kolodziejczyk L., Burnat B. (2016). The effect of Si incorporation on the corrosion resistance of a-C:H:SiOx coatings. Diam. Relat. Mater..

[55] Kaźmierczak T., Niedzielski P., Kaczorowski W. (2020). The influence of plasma-assisted production and milling processes of DLC flakes on their size, composition and chemical structure. Materials.

[56] Thirumalai S., Hausberger A., Lackner J.M., Waldhauser M., Schwartz T. (2016). Effect of the type of elastomeric substrate on the microstructural, surface and tribological characteristics of diamond-like carbon (DLC) coatings. Surf. Coat. Technol..

[57] Sheeja D., Tay B.K., Shi X., Lau S.P., Daniel C., Krishnan S.M., Nung L.N. (2001). Mechanical and tribological characterization of diamond-like carbon coatings on orthopedic materials. Diam. Relat. Mater..

[58] Borowicz P., Latek M., Rzodkiewicz W., Łaszcz A., Czerwinski A., Ratajczak J. (2012). Deep-ultraviolet Raman investigation of silicon oxide: Thin film on silicon substrate versus bulk material. Adv. Nat. Sci. Nanosci. Nanotechnol..

