# Supplementary material for: Barrier Diamond-like Carbon Coatings on Polydimethylsiloxane Substrate
Source: Materials (Basel). 2022 May 29;15(11):3883. doi: 10.3390/ma15113883 (PMC9181918; doi:10.3390/ma15113883)
Supplement: Supplementary file 1 [file materials-15-03883-s001.zip › materials-1725502-supplementary.pdf]

Electronic Supplementary Information

Barrier diamond-like carbon coatings on PDMS substrate

Witold Kaczorowski, Damian Batory, Witold Szymański, Klaudia Lauk and Jakub Stolarczyk

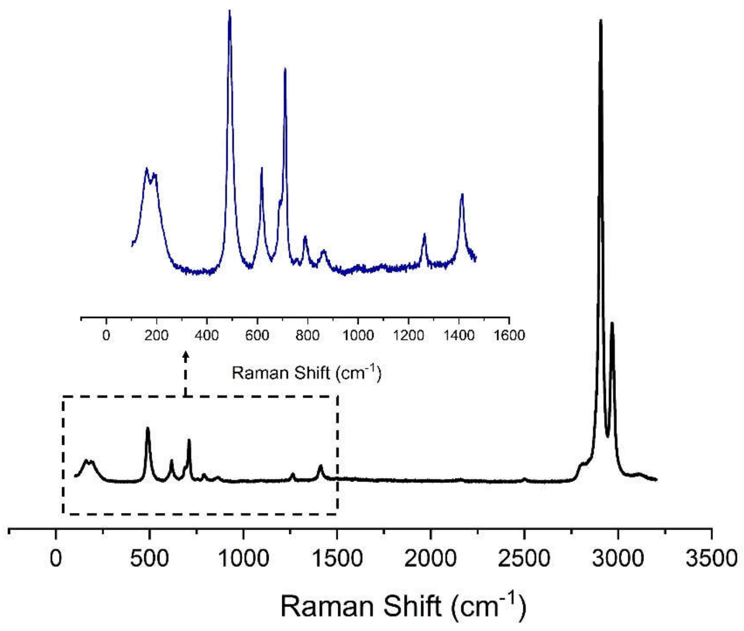

Figure S1: Raman spectra of PDMS substrates

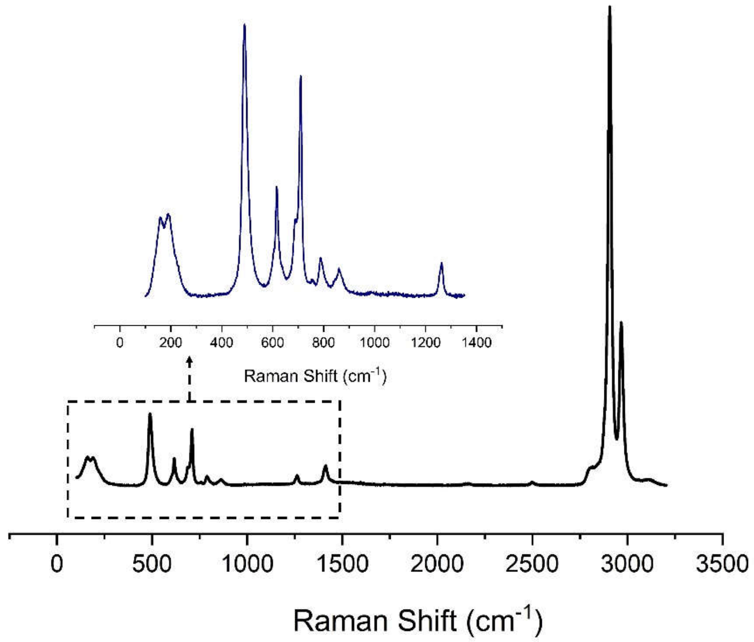

Figure S2: Raman spectra of PDMS substrates modified in argon plasma under the negative self-bias Vb=300V.

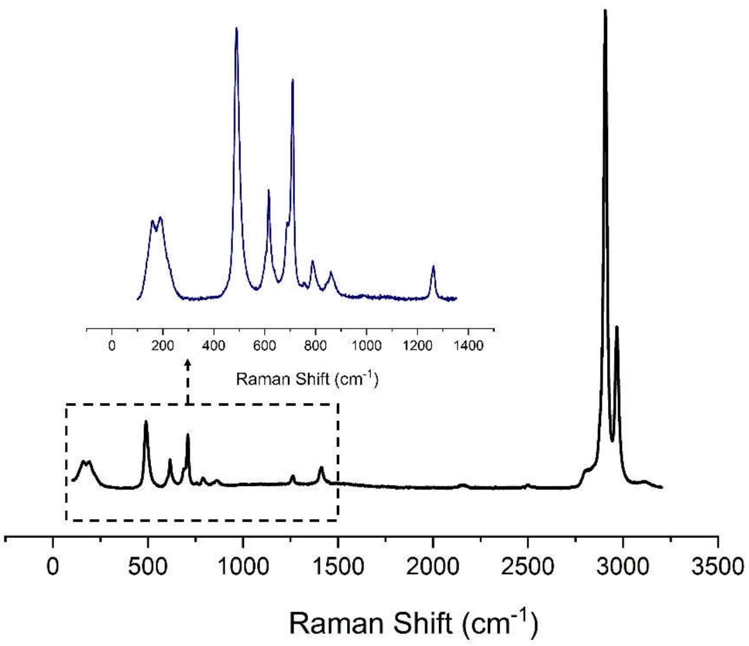

Figure S3: Raman spectra of PDMS substrates modified in nitrogen plasma under the negative self-bias Vb=300V.

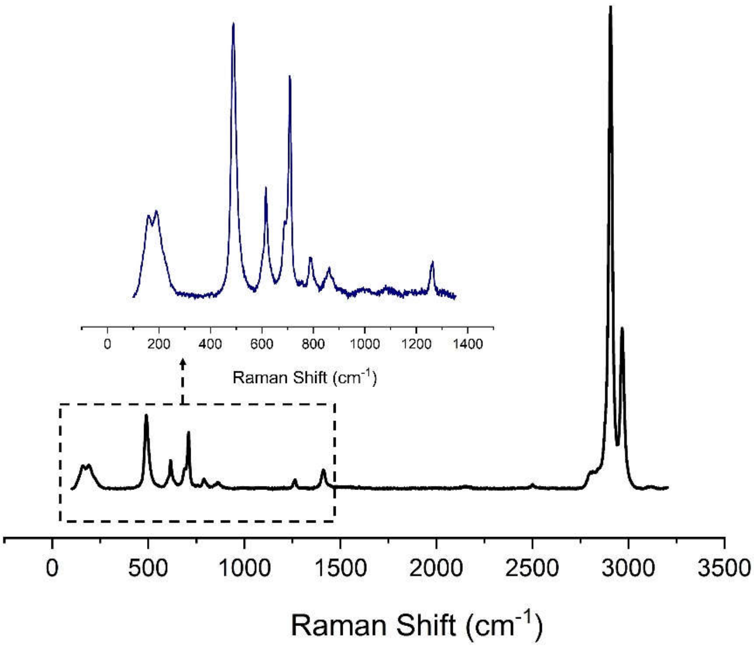

Figure S4: Raman spectra of PDMS substrates modified in oxygen plasma under the negative self-bias Vb=300V.
